# Book Reviews

**Published:** 1893-07

**Authors:** 


### *BOOK REVIEWS.*

---

Diseases of the Skin, their Description, Pathology, Diagnosis and Treatment, with Special Reference to the Skin Eruptions of Children. By H. Radcliffe Crocker, M. D. (Lond.), Fellow of the Royal College of Physicians of London; Physician for Diseases of the Skin in University College Hospital; Late Physician to the East London Hospital for Children; Examiner in Medicine in the Apothecaries' Hall of London. Second edition, revised and enlarged, with 92 woodcuts. Price, $5.00. Philadelphia: P. Blakiston, Son & Co., 1012 Walnut street.

The first edition of this work was published in 1888, and has been exhausted for some time. The present edition, the second, appeared early this year.

The first edition was soon the acknowledged standard, and the second is even better.

The omission of a description of the anatomy of the skin is to be regretted. It should have been included to match the excellent pathological descriptions given throughout the work.

The first edition has received a number of alterations to correspond with the progress of dermatology to date. Besides common diseases of the skin, we find described also, those peculiar to certain localities. The style and type are attractive, the explanations clear and the author's views decided. The matter is so arranged as to render the book easy to be employed either as a manual for beginners or a more full reference book for practitioners.

The illustrations are excellent. Enumeration of the subjects treated would be superfluous. One will find the whole field covered. Medical journals and teachers of dermatology have almost unanimously recommended the book.

The writer has used the first edition for several years as the best work of reference, and the best for the preparation of lectures on dermatology, and indorses the statement of the *New York Journal of Cutaneous and Genito-Urinary Diseases, that this second edition is the best work on skin diseases in the English language.* M. B. H.

AN INTRODUCTION TO THE STUDY OF DISEASES OF THE SKIN. By P. H. Pye-Smith, M. D., F. R. S., F. R. C. P., Physician to the Department of Cutaneous Diseases in Guy's Hospital, London. In one handsome 12mo volume of 407 pages with 28 illustrations, 18 of which are colored. Cloth, $2.00. Philadelphia: Lea Brothers & Co., 1893.

This is a neat little work of about four hundred pages, by an English physician. It is intended simply as a manual, and the writer bases much of the description upon his own experience. He gives various classifications of diseases of the skin, which are as likely to confuse as to instruct the learner. The most common inflammatory diseases are first described, then affections of the "hair-sacs" and skin glands, then "ringworm."

Peculiar features of the book are drawings of the microscopic appearances of diseased skin, the affected part being in a reddish color, and pictures of the naked man upon which is shown, in red, the usual localization of the disease under consideration. These are rather good features, but the localization cannot always be definitely fixed. There is a description of "symptomatic febrile rashes" covering what are usually called the *exanthemata*. Under this head we find erysipelas and the "*roseola syphilitica*." An occasional eruption in cholera and the skin manifestations in influenza are mentioned. He says ringworm of the scroto-femoral region is very hard to cure. A case resisting all other remedies was rapidly cured with half a drachm of pyrogallic acid in an ounce of benzoated lard. (Note: Pyrogallic acid fifteen grains, collodion one ounce was invariably successful in the cases mentioned by the writer of a paper on "ringworm," read before the Atlanta Society of medicine in January, 1892, the writer having accidentally discovered the virtue of the remedy.)

The final chapter is on the "practical classification and diagnosis of diseases of the skin." The usual formulary closes the work.

There seems to be no end to the making of books on skin diseases, both by general practitioners and dermatologists, and nowhere do we see the saying "doctors disagree" better proven than in the mass of these books; of course, however, chiefly in points of classification.

M. B. H.

THE ANATOMY AND SURGICAL TREATMENT OF HERNIA. By Henry O. Marcy, M. D., LL. D., of Boston; Surgeon to the Hospital for Women, Cambridge, Member of the Massachusetts Medical Society, Late President of the American Medical Association, etc. D. Appleton & Co., 1, 3 and 5 Bond Street, New York.

In this book the whole subject of hernia is presented in the most complete and pleasing manner. The distinguished author has devoted much time and labor to the study of this important condition, and his published work will long be one of his rewards. Dr. Marcy believes that there exists a necessity for the better instruction of both surgeon and general practitioner upon the anatomy and surgical treatment of hernia. The illustrations in this volume are excellent. They have been copied from the classic monographs of Astley Cooper, Scarpa and Cloquet, and many beautiful plates have been taken from the works of Longenbeck, Bougery, Cruveilhier, Guthrie, Gay and others, modified and improved by the processes of modern art. The anatomy, pathology and etiology of the different hernias are first clearly and concisely described. One very interesting chapter relates to the development of the truss, and the history of the search for the suitable mechanical support for a reducible hernia. Dr. Marcy warmly advocates the animal suture, and in a special chapter he gives its history, its place in surgery, mode of application, advantages of, etc. But the most important and instructive chapters of all are those which relate to the history and description of the operative measures for the relief of hernia. The methods practiced at the present day are fully discussed. The author's own operation attempts to restore the obliquity of the inguinal canal by the buried tendon suture.

This is a magnificent work which cannot be commended too highly, and those who wish to "read up" on hernia cannot get a better. The mechanical execution of the publishers is in every way excellent and attractive.

---

THE SURGERY AND SURGICAL ANATOMY OF THE EAR. By A. H. Tuttle, M. D. The Physician's Leisure Library. Geo. L. Davis, Detroit.

This little work by Dr. Tuttle is one of the best that has ever appeared in the Leisure Library series. It is a treatise adapted for the aurist and surgeon alike, and although not extensive in the subjects treated, yet it treats such as are of vital importance for satisfactory working in otology. The plates on the anatomy of the ear, which are found in the rear of the book, are photographs from original specimens, and as such give valuable aid to the thorough understanding of the reading portion of the treatise. The author treats of the anatomy of the ear, and then discusses the surgical technique in those diseases requiring such a procedure. The work is valuable in that it brings into a concise form the history of these various surgical procedures up to the present day. Every member of the profession who treats the ear to any extent should possess this valuable little book. C. D. R.

---

MANUAL OF CHEMISTRY. A Guide to Lecturers and Laboratory work for Beginners in Chemistry. A Text-book, specially adapted for Students of Pharmacy and Medicine. By W. Simon, Ph. D., M. D., Professor of Chemistry and Toxicology in the College of Physicians and Surgeons, Baltimore, and Professor of Chemistry in the Maryland College of Pharmacy. New (4th) edition. In one 8vo vol. of 490 pp., with 44 woodcuts and 7 colored plates illustrating 56 of the most important chemical tests. Cloth, $3.25. Philadelphia, Lea Brothers & Co., 1893.

The rapid demand for four editions of this most excellent work is substantial evidence of its value. Dr. Simon has long been a teacher of both medical and pharmaceutic students, and in the preparation of the present work has kept constantly in view the wants of the student. The presence in the volume of seven pages of most beautifully colored plates representing fifty-six chemical reactions is entirely unique and offers most material assistance to the student. Though strictly an elementary work, it is thorough and practical, and we know of no book that will better meet the needs of both teacher and student. L. H. J.

---

APPENDICITIS AND PERITYPHLITIS. By Charles Talamon, M. D., Physician to Tenon Hospital, Paris, France. Physicians' Leisure Library Series. Detroit, Mich.: Geo. S. Davis.

This is a full and complete discussion of this subject in all its bearings by one of the best of the French surgeons. The history of the *lesion*, the causes, symptoms, and its relations to peritonitis are well described, as well as the conditions which may obscure diagnosis. The medical and surgical treatment and operative technique are also described, and the treatment of relapsing cases. Dr. Talamon's little work is a very excellent contribution to the literature of this disease.

---

LESSONS IN PHYSICAL DIAGNOSIS. By Alfred L. Loomis, M. D., LL. D., Professor of the Practice of Medicine and Pathology in the University of the City of New York. Tenth edition, revised and enlarged. Illustrations, some in color. 240 pages, extra muslin, price $3.00. New York: William Wood & Company.

Nearly every practitioner is familiar with Loomis' Physical Diagnosis. The present edition is a thorough revision of this work, and such corrections and additions have been made as seemed necessary to make it a more complete guide to the student. Certain portions have been entirely rewritten. A new chapter has been added, on "Clinical Microscopy." The eminence of the author and the fact that his work has now reached its tenth edition are sufficient to attest the great value of his "Lessons in Physical Diagnosis"

---

INTERNATIONAL CLINICS. A Quarterly of Clinical Lectures. Edited by Drs. John M. Keating, Judson, Daland, J. Mitchell Bruce and David W. Finlay. Third Series, Vol. I. Philadelphia: J. B. Lippincott Co.

Nothing need be said of the current issue of the "Clinics," except that it is in full keeping with its predecessors. The publishers are succeeding in maintaining the excellence and practical character of this work, which is deserving of high commendation.

---

SEXUAL WEAKNESS AND IMPOTENCE. By Edward Martin, M. D., Clinical Professor of Genito-Urinary Diseases, University of Pennsylvania. Physicians' Leisure Library series. Detroit, Mich.: Geo. S. Davis.

In this book Dr. Martin discusses organic, psychical and atonic impotence, with the etiology, diagnosis, prognosis and treatment of each. Chapters are also devoted to prostatorrhœa, involuntary seminal emissions, and impotence in the female.

